# The novel food evaluation process delays access to food innovation in the European Union

**DOI:** 10.1038/s41538-025-00492-x

**Published:** 2025-07-01

**Authors:** Jérôme Le Bloch, Marie Rouault, Cédric Langhi, Malorie Hignard, Victoria Iriantsoa, Olivier Michelet

**Affiliations:** FoodChain ID, 6 rue de la gare, 22000 Saint-Brieuc, France

**Keywords:** Business and industry, Science, technology and society

## Abstract

This article analyzes the timelines of 292 novel food (NF) applications submitted to EFSA between 2018 and 2024 under Regulation (EU) 2015/2283. The average duration from submission to publication was 2.56 ± 1.19 years, with significant variability, and delays due to suitability checks and additional data requests. Improved guidelines and pre-submission support could streamline the process, fostering innovation and timely market access for NFs.

## Introduction

Timely administrative procedures are essential for integrating novel foods (NFs) into current food systems. As society demands more sustainable, functional, and health-promoting food, efficient regulatory frameworks play a pivotal role in facilitating food innovation and timely market access. In the European Union, NFs are defined under Article 3 of Regulation (EU) 2015/2283 as any food not consumed to a significant degree by humans within the Union before 15 May 1997^1^. This regulation, which identifies 10 categories of NFs, also outlines the authorization process. This European regulatory framework for new ingredients has been reviewed recently^2–5^.

The European Food Safety Authority (EFSA) is responsible for the scientific evaluation of the safety of NF applications, with a positive opinion representing a critical milestone for authorization. In brief, the process begins with the submission of a scientific application to the European Commission (EC). The EC starts by verifying the validity of the application without delay and may consult EFSA during this validation phase. EFSA has 30 working days to share its view regarding the validity (Article 6 of Commission Implementing Regulation (EU) 2017/2469). Following verification of the validity of the application, the EC forwards the application for scientific risk assessment to the EFSA without delay (Article 11.1 of Regulation (EU) 2015/2283). EFSA’s primary responsibility in this risk assessment phase is to identify and characterize any potential hazards to human health associated with the novel food, notably compared to similar food already on the EU market, and to assess the risks under the proposed conditions of use (Articles 7 and 11.2 of Regulation (EU) 2015/2283). EFSA is expected to issue its opinion within nine months of receiving a valid application from the EC (Article 11.1 of Regulation (EU) 2015/2283). However, this timeline is almost always extended due to EFSA’s requests for additional information (ADRs) (Article 11.4 of Regulation (EU) 2015/2283), in cases where EFSA deems the initial data provided not sufficient to assess the safety of the NF.

According to EFSA’s 2023 activity report, these delays have significantly impacted the number of evaluations completed, with only 22 assessments finalized in 2023, compared to the target of 42^6^. Following EFSA opinion, the EC and Member States are responsible for the risk management phase. EC prepares the draft implmenting regulation, which is then voted by Member states. EC should present a draft regulation for publication in the Official Journal of the European Union with 7 months.

EFSA’s safety assessment process is recognized as one of the most rigorous globally^7,8^. The scientific requirements for NF applications are detailed in the EFSA’s guidance, updated in 2024^9^ and which specifies that applications must provide evidence of safety for a well-characterized ingredient across ten different sections. EFSA’s ADR frequently pertain to production processes, compositional data, and toxicological assessments, but can cover all sections of the dossier^4^. Recently, two major changes impacted the NF procedure. The implementation of the Transparency Regulation in 2021 (Regulation (EU) 2019/1381), associated with the requirements developed by EFSA in the revised 2021 scientific and technical guidance^10^, has further extended and complicated the NF authorization process. The Transparency Regulation imposes additional regulatory obligations on food business operators prior to submitting an application, particularly concerning the mandatory notification of studies (Article 32b of Regulation (EU) 2019/1381)^11^. Failure to comply with these requirements can result in outright rejection of the application by the authorities, adding another layer of complexity and risk to an already rigorous process. In addition, while the 2021 revised scientific guidance was intended to provide clarity and improve the quality of applications submitted to EFSA, and to reflect the provisions introduced by the Transparency Regulation (EU) 2019/1381^10^, it also introduced complexities for applicants.

EFSA has recently updated its scientific guidance, reflecting the experience it has gained in the assessment of NF applications over the past years^9^. While this 2024 update of the scientific guidance clarifies some of the scientific information needed to prepare and submit an application, it also introduces more stringent requirements for the safety assessment. However, as this guidance is in force since February 1^st^, 2025, its impact on the efficiency of the NF safety evaluation process cannot yet be assessed. Importantly, prolonged or inconsistent evaluation procedures may delay access to safe and beneficial food products, limit consumer choice, and hinder innovation across the food sector.

Despite the importance of this regulatory framework, there is currently no comprehensive evaluation of the timelines involved in the NF application process. A recent analysis of NF applications has suggested an increase in the duration of procedure in the past years^12^, but a detailed analysis of each step of the process has not yet been conducted. Therefore, the objective of this study is to analyze EFSA’s evaluation process for NFs and to quantify the time required for each stage of the process.

## Methods

### Source of information

All data related to NF applications were extracted from publicly available information on the EFSA website^13^, with data collection completed on October 4, 2024, and from the corresponding published EFSA scientific opinions on NFs. The analysis includes only applications submitted under the current Regulation (EU) 2015/2283, which came into effect on January 1, 2018. Applications submitted before this date were not considered in this analysis. Only applications specifically related to NFs authorization were considered, excluding those withdrawn by applicants, terminated by EFSA, or concerning traditional foods from third countries.

### Information analyzed

Applications were classified into four categories based on their status on October 4, 2024^1^: intake (suitability check by EFSA is ongoing)^2^, ongoing risk assessment (scientific evaluation has started), and^3^ adopted (the evaluation is finalized and the opinion has been adopted, this category includes applications marked as ‘publishing’ or ‘published’ on EFSA platform), and^4^ not valid (application rejected by EFSA, in most of the case due to non-compliance with the Transparency Regulation), as presented in Table 1.

**Table 1:** Status of applications according to EFSA website on October 4^th^, 2024

Category	Details	Number of applications
Intake (*i.e*. suitability check)	Applications verified by the EC and received by EFSA, but awaiting suitability check from EFSA are considered as incomplete. Applicants need to complete their application. The scientific evaluation has not yet started.	62
Ongoing risk assessment	Applications valid, and under EFSA scientific evaluation.	107
Adopted	Publishing: EFSA has finished the evaluation and the opinion has been adopted.	2
	Published: EFSA opinion has been published	91
Not valid	Applications rejected by EFSA due to the non-compliance with the transparency regulation. This status concerns only applications submitted Post-Transparency Regulation.	30

Key information extracted for analysis includes: the date of submission of the application to the EC, the date the dossier was received by EFSA, the dossier validation date (the date on which the EFSA suitability check is completed, and validity of the application is confirmed by EC, marking the start of the scientific evaluation), the date of rejection of the application (if applicable), the number and dates of ADRs issued by EFSA, the dates the applicant responded to these ADRs, the date EFSA adopted its opinion, and the date of publication of the opinion. The date of submission of the application and the dates of the ADRs were extracted manually from the published EFSA scientific opinions. The rejection date for applications deemed not valid was extracted manually from the EFSA website^13^. The dossier received date, dossier validated date, opinion adoption date and publication date were extracted automatically from the EFSA website^13^.

**Fig. 1:**
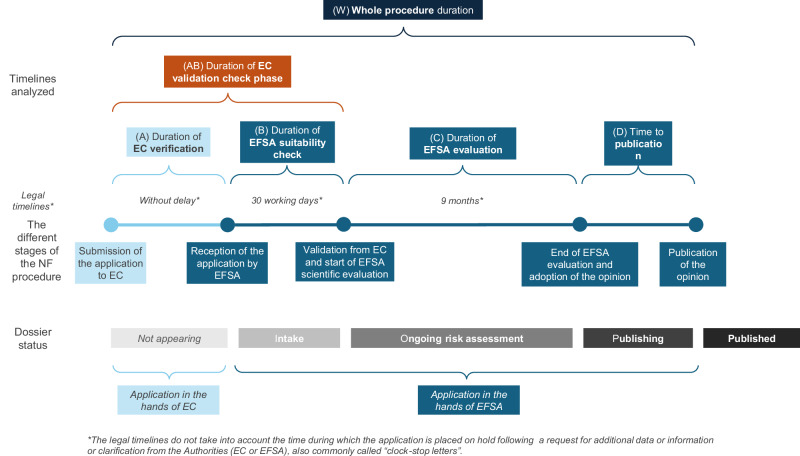
The different stages of the NF procedure and the calculated timelines.

Based on these data points, several timelines were calculated to provide a comprehensive overview of the process (see Table 2 for the definition of each calculated timeline). These calculations include (A) the time taken by the EC to verify the application, (B) the time taken by EFSA to check the suitability and completeness of the application, (AB) the total duration of the validation phase by EC and EFSA (i.e. the time from submission to the start of the scientific evaluation), (C) the duration of the scientific evaluation by EFSA (measured from the validation date to the adoption of EFSA’s opinion), (D) the time from the adoption of the opinion to its publication, and (W) the overall duration of the process encompassing all stages (A, B, C, D) from submission to publication. Figure 1 provides a graphical representation of the different stages of the NF authorization process, and the timelines calculated.

**Table 2:** Definition of the various timelines calculated based on EFSA data

	Timeline analyzed	Definition
(A)	Duration of EC verification	Defined as the difference between the date of submission and the date of reception of the application by EFSA. This is the time needed by EC to verify the receivability of an application.
(B)	Duration of EFSA suitability check	Defined as the time between the reception of the application by EFSA and the date of validation by EC. This also represents the duration EFSA needs to check the suitability/completeness of an application, or “validate” an application.
(AB)	Duration of the EC validation phase	Defined as the difference between the date of submission and the date of validation of the application. This timeline also represents the time to start the EFSA scientific evaluation.
(C)	Duration of EFSA evaluation	Defined as the difference between the date of validation by EFSA and the date of adoption of the opinion. This is the duration of the EFSA scientific evaluation or risk assessment phase.
(D)	Time to publication	Defined as the difference between the date of adoption of the opinion and its date of publication.
(W)	Whole project duration	Defined as the difference between the date of submission of the application and the date of publication of the EFSA opinion.
(E)	Time to rejection	Defined as the difference between the date of reception of the application by EFSA and the date of rejection of the application. This applies only to non-validated applications.

Only applications classified as^2^ ongoing risk assessment^3^, published, and^4^ not valid were included in the timeline analysis. No timeline could be calculated for applications in category^1^ intake, as only the date of receipt by EFSA was available.

For applications rejected by EFSA due to non-compliance with Article 32b of the Transparency Regulation (Regulation (EU) 2019/1381), the (E) timeline, defined as the time from EFSA’s receipt of the application to the date of rejection, was also calculated.

Finally, the final outcome (i.e., positive or negative) of the published opinions was analyzed. Data are reported as mean ± standard deviation.

### Timeline of novel food applications

#### Statistics of the applications on October 4^th^

Table 1 presents the number of applications evaluated in this analysis (*n* = 292), their status on the EFSA platform as of October 4th, 2024, and the category to which they were assigned. Among these, 62 applications were under suitability check (i.e., intake), 107 were undergoing scientific risk assessment, 93 were adopted (i.e., published or publishing), and 30 were rejected as not valid.

**Fig. 2:**
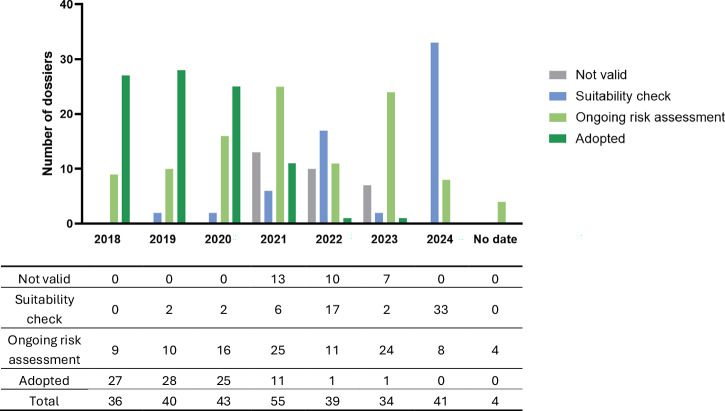
Status on October 4^th^, 2024, of the 292 applications evaluated per year of submission. The date of submission is not available for four applications currently under EFSA evaluation. For some dossiers, the submission year was not available, the submission date was then estimated based on the EFSA question number and NF dossier number.

Figure 2 shows the number of applications submitted per year, categorized by their assigned status as of October 4th, 2024. Overall, 36 applications were submitted in 2018, 40 in 2019, 43 in 2020, 55 in 2021, 39 in 2022, 34 in 2023, and 41 in 2024. The submission date was not available for 4 applications currently under evaluation.

#### Duration of the validation check phase by the European Commission (AB)

A total of 200 applications submitted after 2018 were considered valid by the EC and EFSA, and are now either under scientific evaluation (*n* = 107) or have been finalized (*n* = 93). Among these applications, data was not available for 4 applications, while two applications related to insects were identified as outlier due to abnormally long EC validation times, and were excluded from the analysis (applications NF 2018/0395 and NF 2018/0128, 1620 and 2296 days for validation respectively). Consequently, data from 194 applications were included in the analysis.

The average time needed for the EC to verify the application after submission is (A) 114 ± 181 days (range: 0–1430 days). The mean length of EFSA suitability check is (B) 185 ± 122 days (range 15–758 days). The average duration of the whole validation check phase, defined as the total time between the submission of the application and the start of its evaluation, is (AB) 299 ± 218 days (range 20–1635 days).

#### Duration of the scientific evaluation of the applications by EFSA (C)

EFSA has published or is publishing 93 NF opinions for applications submitted since 2018. However, the date of validation is not available for 5 applications. For the remaining 88 opinions, average duration of EFSA evaluation was (C) 629 ± 338 days (i.e. 20.7 ± 11.1 months). The shortest evaluation lasted 179 days (i.e. 5.9 months), while the longest one lasted 1714 days (i.e. 56.4 months).

The average number of ADRs from EFSA is 2.7 ± 1.9, ranging from 0 to 8 requests. Applicants need 130 days on average to answer one EFSA ADR (range 0–733 days). The overall cumulated time needed to answer all EFSA ADRs is 353 ± 299 days (range 0–1213 days). The time taken by applicants to answer EFSA requests represents 47 ± 25% of the total evaluation time, while EFSA evaluation represents only 53 ± 25% of the total time needed for evaluation.

Among these 93 applications published or publishing, EFSA exceeded the standard nine-month evaluation period for 24 applications, representing 26% of the applications. The average overrun is 156 ± 212 days (range: 5–951 days). It should be noted, however, that Article 22 of Regulation (EU) 2015/2283 authorizes the EC to extend the time period for evaluation.

#### Time to publication (D)

For the 91 published EFSA outputs, the average time from the vote of the Nutrition, Novel Foods, and Food Allergens Panel to the publication of the opinion (D) was 48 ± 16 days, with a range of 26 to 107 days.

Among these 91 EFSA opinions evaluated at the time of data extraction, the safety of the novel food was confirmed in 79 cases, while 12 applications were rejected following evaluation. This corresponds to a positive opinion rate of 86.81%.

The main reasons for rejection were safety concerns (*n* = 8). Other reasons included the inability to identify the source of the novel food (*n* = 1) and poor application quality, including the lack of pertinent responses from the applicant (*n* = 3).

#### Duration of whole procedure timeline (W)

Overall, the average time from dossier submission to EFSA’s adoption of the opinion (AB + C) was 889 ± 433 days (range: 289–2255 days). The total time from submission to publication of EFSA opinion is (W) averaged 937 ± 436 days (range: 330–2314 days), which corresponds to approximately 31 ± 14 months, or 2.56 ± 1.19 years.

**Fig. 3:**
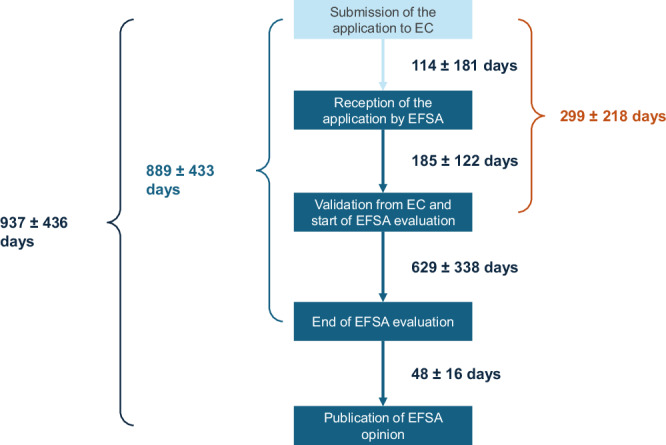
Analysed timelines of NF applications in Europe, from submission to the publication of EFSA opinion. Numbers represent mean ± SD.

Figure 3 illustrates the timeline of NF applications in Europe, from submission to the publication of EFSA opinion.

#### Applications rejected by EFSA due to non-compliance with the transparency regulation

Thirty applications were rejected by EFSA due to non-compliance with Article 32b of the Transparency Regulation, which mandates prior notification of studies. As this regulation came into force on March 27, 2021, only applications submitted after that date were affected (13 applications in 2021, 10 in 2022 and 7 in 2023). Among these, only two applications had entered the scientific evaluation phase when they were deemed not valid; the remaining 28 applications were still in the suitability check phase (i.e. intake) at the time of rejection.

The average time to rejection, defined as the period between EFSA’s receipt of the application and its rejection (E) was 297 ± 121 days (i.e. 10.6 ± 4.3 months), with a minimum of 74 days (2.6 month) and a maximum of 570 days (20.4 months). A slight decrease in the time needed to reject the application is noted (334 ± 90 days for applications submitted in 2021, 308 ± 132 days for those submitted in 2022, and 214 ± 130 days for those submitted in 2023).

## Conclusion

Our analysis presents the first comprehensive analysis of the processing timelines for NF applications under Regulation (EU) 2015/2283, shedding light on procedural inefficiencies and administrative bottlenecks within the EU’s regulatory framework. Our findings reveal substantial variability at each stage of the authorization process, with an average duration of 2.6 ± 1.2 years from submission to publication, and outliers extending up to six years. Administrative steps, including EFSA’s suitability check and EC validation, account for nearly a year, but show significant variability, likely due to inconsistent dossier quality and procedural inefficiencies. During the scientific evaluation phase, EFSA frequently issues ADRs, which contributes to extended timelines and represents nearly half of the total evaluation time. While these ADRs often stem from missing or incomplete information required to assess NF safety, they may also reflect EFSA’s precautionary approach, consistent with its mandate to ensure high standards of food safety in the EU. Despite these procedural challenges, 86.81% of EFSA’s opinions are ultimately positive, underscoring the robust performance of the regulatory system in supporting the safe introduction of novel foods into the EU market.

These findings carry important implications for policymakers, industry stakeholders, consumers and society. While this analysis does not question the necessity of the NF authorization process or EFSA’s role in ensuring food safety, the lengthy administrative procedures, despite a high rate of positive EFSA opinions, suggest that the current framework may be excessively burdensome. Such inefficiencies could discourage applicants from engaging with the regulatory process and risk undermining the innovation-friendly environment that the European Union aims to promote. In some cases, prolonged evaluations may contribute to the failure of food businesses, including those receiving public funding, by delaying market access beyond sustainable timelines. Moreover, these delays may limit consumer access to safe and innovative food products, at a time when global challenges, such as sustainability, economic competitiveness, and evolving dietary needs, require prompt and effective solutions.

EU policies on sustainable food systems, most notably the *Farm to Fork Strategy*, a central component of the European Green Deal, aim to make food systems more fair, healthy and environmentally-friendly^14^. Achieving sustainable food production necessitates the development and approval of new food sources, with NFs playing a crucial role in the realization of resilient and sustainable food systems^3,15,16^. Recent studies, such as that by Mazac *et al*., have demonstrated the significant environmental benefits of integrating NFs into whole diets. Replacing conventional animal proteins with plant-based and NF alternatives can reduce global warming potential, scarcity-weighted water use, and land use by over 80%^17^. However, while these sustainable alternatives, including plant-based proteins, fungal biomasses, and cellular meat, are already approved in other regions, they remain largely inaccessible in the EU due to pending regulatory approvals. This regulatory lag risks undermining both the competitiveness of the European food industry and its ability to adapt to evolving societal demands. In parallel, the valorization of agri-food waste represents a promising avenue to create functional and sustainable NFs, contributing to the reduction of food waste and environmental impact^3,16^. Yet, despite their potential to reduce environmental impacts, the authorization of new food ingredients presents a significant obstacle, as illustrated for instance by the use of coffee by-products^18^, potentially impeding innovation and the rapid adoption of eco-friendly practices across the food sector.

The European NF process may represent a significant barrier to market entry. The NF regulation has been shown hampers entrepreneurial experimentation, resource mobilization, investments and market formation^12,19^. The current pre-market approval process has been recognized as a disincentive for innovation and competitiveness among food business operators^20,21^. The EU regulatory landscape is widely perceived as challenging, particularly due to lengthy authorization timelines and the lack of effective dialogue between stakeholders and regulatory authorities^22^. Our analysis confirms this perception: the significant duration of NF approvals is likely to be viewed by investors and innovative companies as a major obstacle to the development of new ingredients^12^.

Importantly, while EFSA’s role in risk assessment is essential for protecting consumers, its involvement is not mandatory under Article 11 of Regulation (EU) 2015/2283. EFSA is only required to evaluate an application when requested by the European Commission. In practice, however, the EC systematically refers applications to EFSA, even in cases where a scientific opinion may not be strictly necessary—such as when the ingredient has been previously assessed or involves only minor modifications. Implementing more robust upstream risk assessments could streamline the process by reducing the number of dossiers sent to EFSA, allowing the agency to concentrate on more complex or novel evaluations.

Taken together, our findings underscore the need for a critical reassessment of the procedural design of NF evaluations in the EU. Balancing scientific rigor with regulatory efficiency is essential to maintaining food safety without impeding innovation. EFSA could maintain its high safety standards while improving efficiency, for instance, through clearer guidance, enhanced dossier quality, and more structured pre-submission dialogue. Such measures would help reduce approval timelines and facilitate broader access to safe, innovative food products across the European market.

## Supplementary information

Article_J.Lebloch_Dataset

## Data Availability

Raw data is available in the supplementary files.

## Abbreviations

ADR: Additional data request
EFSA: European Food Safety Authority
EC: European Commission
NF: Novel Food
